# YOLO-TPS: A Multi-Module Synergistic High-Precision Fish-Disease Detection Model for Complex Aquaculture Environments

**DOI:** 10.3390/ani15162356

**Published:** 2025-08-11

**Authors:** Cheng Ouyang, Hao Peng, Mingyu Tan, Lin Yang, Jingtao Deng, Pin Jiang, Wenwu Hu, Yi Wang

**Affiliations:** 1 College of Information and Intelligence, Hunan Agricultural University, Changsha 410128, China; ouyang@stu.hunau.edu.cn (C.O.); sx20230201@stu.hunau.edu.cn (H.P.); 19907426171@stu.hunau.edu.cn (M.T.); 2767959117@stu.hunau.edu.cn (L.Y.); 984711035@stu.hunau.edu.cn (J.D.); 2 College of Mechanical and Electrical Engineering, Hunan Agricultural University, Changsha 410128, China; 1233032@hunau.edu.cn

**Keywords:** deep learning, fish disease recognition, YOLOv11n, multi-module synergistic, aquaculture, animal health monitoring

## Abstract

Fish are a vital aquatic resource worldwide, and the sustainable development of aquaculture is essential for global food security and economic growth. However, the high incidence of fish diseases in complex aquaculture environments significantly hampers sustainability, and traditional manual diagnosis methods are inefficient and often inaccurate. To address the challenges of small-lesion detection, lesion area size and morphological variation, and background complexity, we propose YOLO-TPS, a high-precision fish-disease detection model based on an improved YOLOv11n architecture. The model integrates a multi-module synergy strategy and a triple-attention mechanism to enhance detection performance. Specifically, the SPPF_TSFA module is introduced into the backbone to fuse spatial, channel, and neuron-level attention for better multi-scale feature extraction of early-stage lesions. A PC_Shuffleblock module incorporating asymmetric pinwheel-shaped convolutions is embedded in the detection head to improve spatial awareness and texture modeling under complex visual conditions. Additionally, a scale-aware dynamic intersection over union (SDIoU) loss function was designed to accommodate changes in the scale and morphology of lesions at different stages of the disease. Experimental results on a dataset comprising 4596 images across six fish-disease categories demonstrate superior performance (mAP_0.5_: 97.2%, Precision: 97.9%, Recall: 95.1%) compared to the baseline. This study offers a robust, scalable solution for intelligent fish-disease diagnosis and has promising implications for sustainable aquaculture and animal health monitoring.

## Simple Summary

Fish farming is crucial for global food security, but diseases in complex underwater environments threaten its sustainability. This study developed a new technology, called YOLO-TPS, to detect fish diseases accurately and early, addressing challenges like small disease signs, changing symptoms, and murky water. The goal was to create a smart system to identify diseases quickly, reducing losses and the need for antibiotics. Using a dataset of 4596 images covering six fish-disease types, the model was trained to recognize issues like ulcers and parasites. Results showed it achieved a 97.2% accuracy rate, significantly better than existing methods, and excelled at spotting small disease signs in challenging conditions. The technology helps farmers catch diseases early, improving fish health and reducing environmental harm from treatments. By offering a reliable, efficient tool, this work supports sustainable fish farming, protects food supplies, and promotes healthier aquatic ecosystems. Its approach could also be applied to other animal- or plant- disease detection, benefiting agriculture and environmental management globally.

## 1. Introduction

Fish farming plays a vital role in global food security and rural economic development, but the increasing incidence of infectious diseases poses a major threat to the sustainability of aquaculture systems. Disease outbreaks not only reduce productivity and profitability, but also lead to over-reliance on antibiotics, raising serious concerns about drug resistance and environmental impact. In intensive aquaculture settings, early and accurate detection of fish diseases is essential for improving animal welfare, minimizing losses, and supporting sustainable farm management. Therefore, developing a high-precision, automated fish-disease detection model is of significant importance for enhancing farming efficiency, reducing resource waste, and promoting the establishment of environmentally friendly and sustainable aquaculture systems.

Traditional fish-disease detection primarily relies on manual visual inspection, which is time-consuming, costly, and highly dependent on the subjective expertise of diagnosticians, making it inadequate for the efficiency and precision required by modern aquaculture. Recent advances in computer vision and deep learning technologies have provided a powerful foundation for intelligent disease diagnosis. Convolutional Neural Networks (CNNs), in particular, have demonstrated significant potential in the detection of diseases in both animals and plants. For instance, Cuan et al. [1] utilized CNNs to analyze chicken vocalization data, achieving precise identification of avian influenza infections; Xu et al. [2] integrated depth imaging with a CNN-SVM model to develop an automated system for group-level pig posture scoring; Qian et al. [3] enhanced CNN models by introducing self-attention mechanisms, improving the accuracy of corn-leaf disease identification. Moreover, Pandian et al. [4], Vishnoi et al. [5], and Gao et al. [6] achieved a balance between high accuracy and computational efficiency in plant-leaf disease detection, by optimizing CNN architectures.

The incorporation of attention mechanisms has further improved the ability of deep learning models to focus on critical lesion regions, thereby enhancing both accuracy and robustness in disease identification. Stephen et al. [7] developed a self-attention-based ResNet architecture for rice-leaf disease classification, significantly improving the model’s feature extraction capabilities. Shinde et al. [8] combined the VGG16 CNN architecture with attention mechanisms to achieve efficient detection of bovine diseases. Zhang et al. [9] utilized thermal infrared imaging and attention-enhanced CLEUNet to detect bovine mastitis, with high precision. Additionally, Zhang et al. [10] proposed a YOLOv5 model incorporating coordinate attention mechanisms, coupled with quantum-dot fluorescence biosensing technology, enabling rapid detection of poultry diseases. Numerous studies have further validated the advantages of attention mechanisms in plant- and animal-disease detection tasks, including tea plants, dairy cows, and poultry [11,12,13,14,15,16,17], by enhancing key feature extraction and suppressing irrelevant information, thereby improving model adaptability across diverse application scenarios.

It is noteworthy that in the domain of fish-disease recognition, the YOLO (You Only Look Once) series of object detection models have been widely adopted in intelligent aquaculture research, due to their efficiency and real-time performance. Liang et al. [18] utilized visual sensors and applied image recognition and tracking technologies to monitor the physiological status of fish. Wang et al. [19] proposed an enhanced YOLOv5 model combined with the SiamRPN++ algorithm to achieve real-time detection and tracking of abnormal fish behavior. Wang et al. [20] further optimized the YOLOv5 network for diseased fish detection in underwater environments, significantly improving model performance. Yu et al. [21], Medina et al. [22], and Cai et al. [23] achieved efficient detection of various fish skin diseases using YOLOv4, YOLO, and YOLOv7, respectively. Furthermore, Hwang et al. [24], Jiang et al. [25], Li et al. [26], Tang et al. [27], and Zhou et al. [28] achieved superior detection performance by refining YOLO architectures for specific tasks, including flatfish-disease diagnosis, big data analysis in fish pathology, multi-task learning, and Cryptocaryoniasis identification. Most recently, Wang et al. [29] leveraged an improved YOLOv10 model to enhance real-time detection and recognition of fish skin health in underwater settings, markedly boosting model robustness under occlusion and complex background conditions.

Despite significant advancements in fish-disease detection technologies, unique challenges inherent to aquaculture environments continue to impose stringent demands on existing methodologies. First, underwater imaging is often compromised by uneven illumination (e.g., strong reflections or low-light conditions) and complex backgrounds, including water turbidity, algae, and debris, all of which degrade image quality and increase the difficulty of feature extraction. These factors elevate the risk of model misclassification. Second, the external manifestations of fish diseases vary considerably in morphology and scale throughout the disease progression. For instance, early-stage lesions—such as small parasitic infestations or initial ulcerations—typically present as low-contrast, small-scale targets, whereas advanced infections may result in large, cotton-like lesions or deep ulcerations. Such dynamic variations necessitate detection models possessing robust multi-scale adaptability to accurately identify a wide spectrum of lesion types. Third, early-stage lesions are often subtle, small in size, and low in contrast, making them easily obscured by background noise, which leads to elevated rates of missed or false detections by conventional models during early diagnosis. These challenges are particularly pronounced in aquaculture settings, where underwater imaging is affected by factors such as light refraction, water quality fluctuations, and the constant movement of fish. Compared to terrestrial disease detection tasks (e.g., on plant leaves or poultry surfaces), underwater disease recognition involves greater complexity and uncertainty. Furthermore, the rapid movement of fish and dynamic occlusion in aquaculture environments exacerbate the discontinuity of target features in sequential image frames, thereby imposing higher requirements on the real-time performance and robustness of detection models. To address these challenges, this study proposes an advanced detection method based on the YOLOv11n architecture, designed to enhance model performance under the complex and dynamic conditions of real-world aquaculture.

The approach integrates the SPPF_TSFA module to enhance multi-scale feature extraction, combines the Shuffleblock module with windmill-shaped convolution (PConv) to improve spatial awareness, and replaces the CIoU loss function with a Scale-Dynamic IoU (SDIoU) loss to optimize detection accuracy, particularly in scenarios with uneven illumination and complex backgrounds. The paper is organized as follows: Section 2 details the dataset acquisition and integration, as well as the construction of the proposed YOLOv11n-based model. Section 3 presents the experimental results, including ablation studies and comparative analyses. Section 4 discusses the model enhancements, performance robustness, and dataset optimization strategies. Section 5 concludes with a summary of the findings and the model’s potential for practical applications in aquaculture.

## 2. Materials and Methods

### 2.1. Datasets

#### 2.1.1. Overview of the Datasets

In this study, a comprehensive fish-disease image dataset was constructed by integrating a self-collected fish image dataset with a publicly available dataset from the Roboflow platform (Fish Skin Diseases Detection) [30]. The self-collected dataset focuses on healthy fish and those infected with Saprolegniasis, aiming to provide high-quality, localized data for the accurate detection of this specific disease. In contrast, the public dataset encompasses a broader spectrum of fish skin conditions, including Columnaris Disease, Epizootic Ulcerative Syndrome, Gill Disease, and Streptococcus Disease, as well as healthy specimens. The inclusion of this dataset significantly enhances both the pathological diversity of the overall dataset and the generalization capability of the resulting models. The final integrated dataset constitutes a multi-class, balanced image collection, well-suited for the training and evaluation of deep learning models.

#### 2.1.2. Self-Collected Dataset Acquisition

The images used to construct the self-collected dataset were acquired in 2024 at the Zongnan standardized aquaculture facility in Jishou, Xiangxi, Hunan Province, China, with the primary focus on cultured fish species. To address the complexity inherent in fish-disease identification, the dataset was designed to encompass a wide range of representative scenarios. These include images captured under varying lighting conditions, samples containing multiple disease types within a single image, and images featuring either individual or multiple diseased fish. All images were captured using smartphones from multiple angles to ensure high-resolution imaging and the preservation of fine pathological details. Image acquisition was conducted on the indoor flooring of the aquaculture facility, where factors such as surface reflections, uneven lighting, and complex backgrounds posed additional challenges. Detailed acquisition conditions are summarized in Table 1.

To address challenges associated with image acquisition, including blurriness, lighting anomalies, and occlusion of target objects, a comprehensive quality control strategy was developed in this study. This strategy was complemented by manual inspection to systematically screen and eliminate images exhibiting severe blur, overexposure, underexposure, or significant fish body overlap that could impair diagnostic accuracy. The specific procedures are summarized in Table 2. Initially, each image was manually reviewed, and those suffering from substantial blurring, lighting defects, or occlusions due to overlapping fish were discarded. Blurriness was quantitatively assessed using the Laplacian operator to compute edge gradients; images with a variance below 100 were classified as severely blurred, and removed. Lighting anomalies were identified via histogram equalization analysis, and images with mean brightness values outside the range of [50, 200] were excluded. In multi-fish overlap scenarios, only images where at least 50% of a fish’s body contour was clearly visible were retained.

To ensure annotation accuracy, all remaining images underwent pixel-level labeling using the LabelMe tool. The annotation process was independently conducted by two trained experts, followed by cross-validation. The inter-annotator agreement, measured by the Kappa coefficient, exceeded 0.85, ensuring a high degree of correspondence between class labels and pathological features. By rigorously applying the aforementioned filtering criteria, a total of 534 high-quality images were retained from the original dataset. These images provided a clear, stable, and diagnostically informative foundation for subsequent model training, thereby enhancing both the overall utility of the dataset and the robustness of model performance.

**Figure 1:**
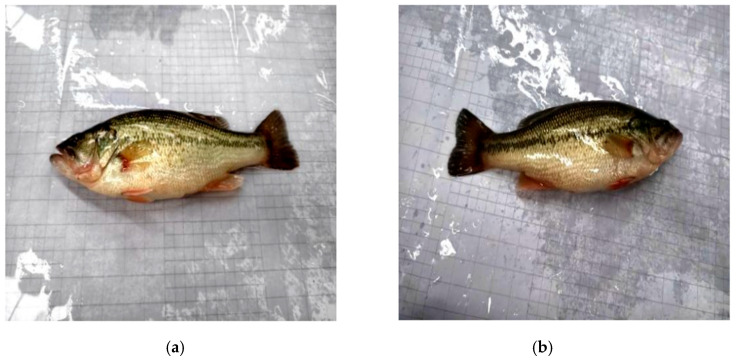
Healthy Fish Image Presentation: Image (**a**) on the left displays the complete fish contour, while image (**b**) on the right highlights skin texture and color distribution, serving as a stable reference for healthy fish features in deep learning models.

In this study, a total of 245 images of healthy fish were collected, all captured from a lateral perspective to fully present the body contours and external morphology, as illustrated in Figure 1. This viewpoint was selected not only to minimize the impact of postural variations on feature extraction, but also to facilitate accurate capture of the skin texture and color distribution patterns of healthy fish. Such consistency in imaging provides a stable and uniform foundation for subsequent classification tasks.

**Figure 2:**
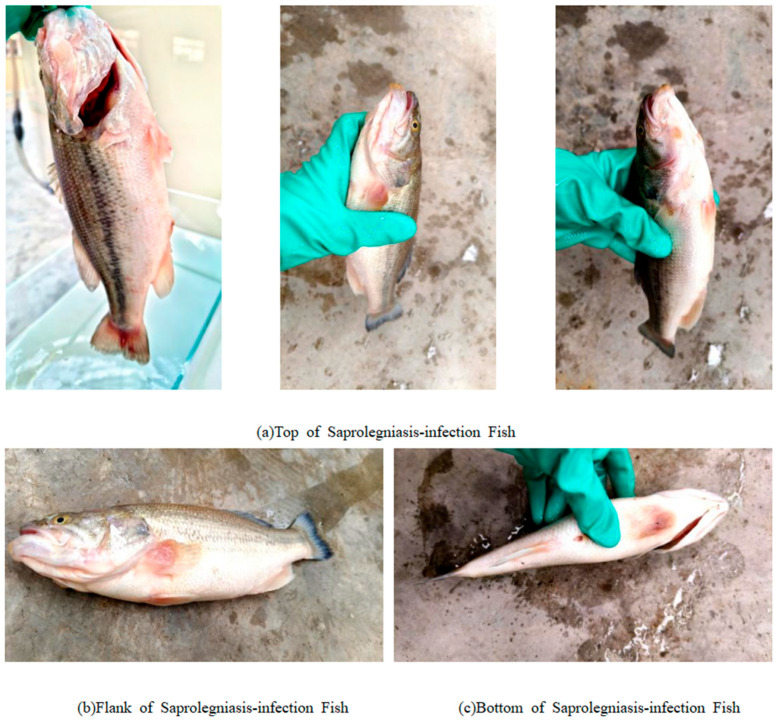
Saprolegniasis-infection Fish Image Presentation: Image (**a**) shows white cotton-like mycelium on the dorsal surface, (**b**) highlights lesion distribution and fin damage from a lateral view, and (**c**) displays mycelium attachment on the ventral surface, capturing diverse phenotypic features to enhance model training diversity.

A total of 289 images of fish affected by Saprolegniasis were collected, encompassing three distinct perspectives: lateral, dorsal, and ventral views. This multi-angle acquisition strategy was designed to comprehensively capture the characteristic pathological features of Saprolegniasis across different anatomical regions of the fish, such as the presence of white, cotton-like mycelial growths, as illustrated in Figure 2. The incorporation of multiple viewpoints significantly enhances the phenotypic diversity of the dataset, thereby facilitating the deep learning model’s ability to learn and generalize the complex morphological manifestations of the disease.

#### 2.1.3. Public Dataset Introduction

**Figure 3:**
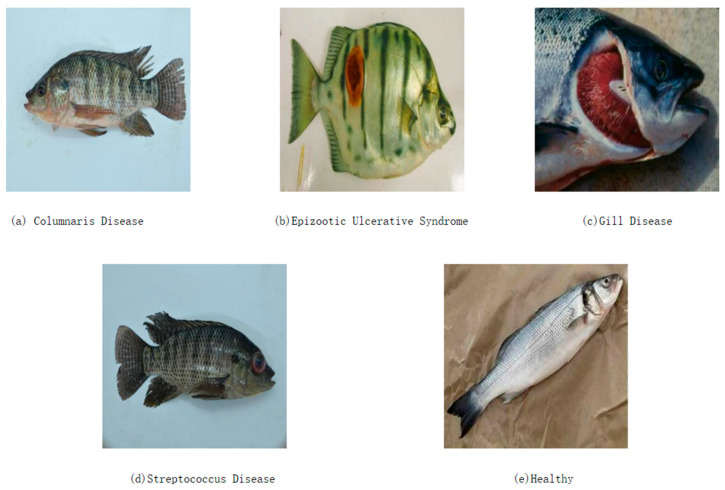
Public dataset image presentation: Image (**a**) shows white patches indicative of Columnaris Disease, (**b**) displays deep skin ulcers characteristic of Epizootic Ulcerative Syndrome (EUS), (**c**) highlights gill tissue necrosis in Gill Disease, (**d**) presents ocular opacity and skin erythema in Streptococcus Disease, and (**e**) shows intact skin and smooth scales in healthy fish.

To further enhance the pathological diversity of the dataset, this study incorporated the publicly available Fish Skin Diseases Detection dataset provided by the Roboflow platform. This dataset encompasses five classes: Columnaris Disease, Epizootic Ulcerative Syndrome (EUS), Gill Disease, Streptococcus Disease, and Healthy Fish, comprising a total of 3528 images, as illustrated in Figure 3. The dataset has been pre-divided into training (3087 images, 88%), validation (294 images, 8%), and test (147 images, 4%) subsets, according to scientifically appropriate ratios, thereby ensuring a rational data distribution for model development and performance evaluation in deep learning tasks.

Regarding data augmentation, each training sample in this dataset was used to generate three augmented outputs. The augmentation process included random rotation within the range of −11° to +11° to simulate various postures of fish in natural aquatic environments. Saturation was varied within ±25%, and brightness was adjusted within ±15%, effectively simulating variations in lighting conditions and water quality that may influence image appearance. To maintain consistency between annotated labels and transformed images, identical transformations—including rotation within −11° to +11° and brightness adjustment within ±15%—were applied to the bounding boxes. These augmentations collectively provide more challenging and realistic samples, thereby improving the robustness and generalizability of the trained models.

### 2.2. Data Preprocessing

#### 2.2.1. Data Augmentation

**Figure 4:**
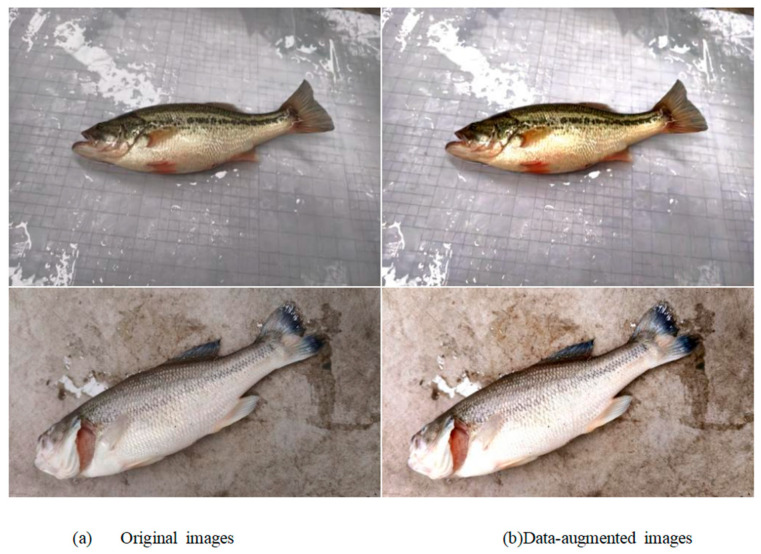
Data-Augmentation Presentation.

To ensure the quality of the self-collected dataset and effectively expand the sample size to support the training of a high-precision fish-disease recognition model, a systematic data preprocessing and augmentation strategy was designed and implemented in this study. The diversification of training samples not only enhanced the model’s ability to learn and recognize critical pathological features, but also significantly improved its generalization performance and robustness under complex environmental conditions [31,32,33]. Considering the specific imaging characteristics of the self-collected data, a color jitter–based augmentation approach was employed. By adjusting brightness, contrast, and color saturation—each with an augmentation factor range of [0.8, 1.2]—this method simulated variations in illumination and background conditions. Additionally, geometric transformations were applied to further enrich data diversity. A total of 245 images of healthy fish and 289 images of fish affected by Saprolegniasis were collected. The images were captured in an indoor aquaculture facility under conditions of uneven lighting and complex backgrounds. Representative augmented images are shown in Figure 4.

The images were annotated at the pixel level using the LabelMe tool, with class labels designated as “Healthy” and “Saprolegniasis”. To meet the input requirements of the YOLOv11 model and to manage computational costs, all images were resized to 640 × 640 pixels via bilinear interpolation. This resizing ensured the preservation of essential features, while maintaining consistency with the publicly available Fish Skin Diseases Detection dataset. Following preprocessing and data augmentation, the total number of self-collected images increased from 534 to 1068, as shown in Table 3. This process adjusted the ratio of healthy to Saprolegniasis-infected samples to approximately 1:1.24, thereby mitigating the issue of class imbalance. All augmented images were manually reviewed to ensure that pathological features remained intact and were not distorted by the applied transformations. This augmentation significantly enhanced the dataset in terms of both scale and diversity.

#### 2.2.2. Dataset Integration

To establish a unified dataset tailored for multi-class fish-disease identification, this study systematically integrated a self-collected dataset (comprising 1068 images) with a publicly available dataset (Fish Skin Diseases Detection, containing 3528 images), resulting in a comprehensive dataset of 4596 images. During the integration process, class labels were first standardized by mapping the self-collected dataset’s “Healthy” and “Saprolegnia infection” categories onto the five existing classes in the public dataset—namely, healthy fish, columnaris disease, ulcerative syndrome, gill disease, and streptococcosis—thereby forming a six-class labeling scheme: healthy, Saprolegnia infection, columnaris disease, ulcerative syndrome, gill disease, and streptococcosis. To ensure annotation format consistency, bounding box annotations from the self-collected images were converted to the YOLO format to align with the public dataset’s labeling protocol. Detailed composition statistics are presented in Table 4. The unified dataset was then partitioned into training and testing subsets, with an 80:20 split, comprising 3676 and 920 images respectively, to guarantee the scientific rigor and representativeness of model training and evaluation. The resulting dataset exhibits a relatively balanced class distribution, wherein the self-collected images contribute abundant samples for healthy and Saprolegnia-infected fish, while the public dataset enriches the dataset with diverse pathological features, thereby enhancing the model’s generalization capability and robustness.

### 2.3. Fish-Disease Classification

#### 2.3.1. YOLO-TPS Network Construction

As the latest evolution in the YOLO series [34], YOLOv11 builds upon the highly efficient architecture of its predecessors, further enhancing detection accuracy and inference speed. It has been widely adopted in real-time object detection tasks. The Backbone employs an improved CSPNeXt architecture, which integrates the Cross-Stage Partial (CSP) mechanism with lightweight convolutional modules. This design effectively reduces parameter count and computational overhead, while preserving strong feature-representation capabilities, significantly improving robustness in detecting small targets and objects within complex backgrounds. The Neck incorporates an enhanced SPPFCSPC structure combined with a Bi-directional Feature Pyramid Network (BiFPN), which, through a multi-scale fusion strategy, strengthens feature interaction across semantic layers and improves recognition performance for scale-variant targets. In the Head, YOLOv11 utilizes a decoupled detection head that models classification, regression, and object confidence tasks separately, enabling more refined feature representations and more stable gradient propagation, thereby boosting overall detection accuracy and training convergence speed.

**Figure 5:**
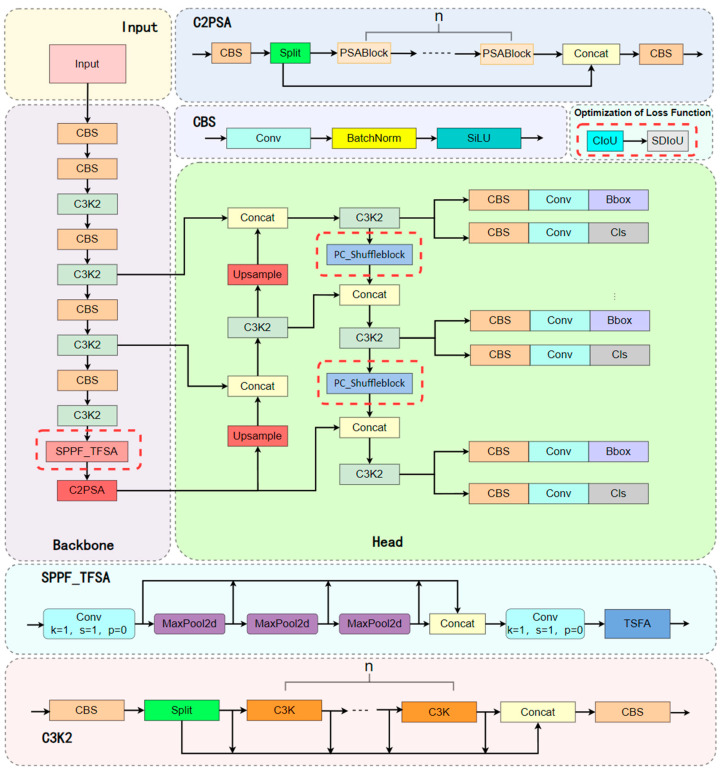
YOLO-TPS network structure diagram.

In the context of fish-disease detection, where challenges such as uneven lighting, complex backgrounds, dynamic disease characteristics, and small-lesion features prevail in aquaculture scenarios [35,36,37], this study introduces a series of optimizations based on the YOLOv11n model. Firstly, within the Backbone, a novel SPPF_TSFA module was designed to further enhance multi-scale feature representation. The Tri-Level Selective Focus Attention (TSFA) mechanism, operating at spatial, channel, and neuron levels, strengthens the feature expression of critical lesion areas, markedly improving detection accuracy for small-scale disease spots. Secondly, in the Head, the PC_Shuffleblock module was incorporated by replacing the 1 × 1 convolution in ShuffleBlock with Pinwheel Convolution (PConv), thereby enhancing spatial awareness of low-level features and increasing target response sensitivity. The PConv module employs asymmetrical convolution kernels to expand the receptive field while reducing parameter overhead, thus bolstering the model’s capacity to capture complex textures and edge details. Finally, addressing limitations of the conventional CIoU loss function, this work adopts a Scale-Dynamic IoU (SDIoU) loss function tailored to accommodate the dynamic progression of fish-disease symptoms. By introducing a scale-dynamic regulation factor, SDIoU adaptively balances scale loss and localization loss weights, enhancing the model’s adaptability to small-scale lesions and fuzzy boundaries, ultimately reducing false negatives and false positives and improving detection performance. Key modules modified within the network are highlighted by red dashed boxes, and the structure of the improved network model is illustrated in Figure 5.

#### 2.3.2. Application of a Tri-Level Selective-Focus-Attention Fusion Mechanism to Deep Feature Enhancement

In the automatic identification of fish-disease images, especially for early-stage lesions such as initial ulcers and micro-parasitic infections, the small size and low contrast of target regions often result in significant missed detections and false positives by detection models, severely compromising the accuracy of early diagnosis. To address these challenges, this study proposes a multi-scale feature enhancement architecture that integrates a Tri-Level Selective Focus Attention (TSFA) module with a Spatial Pyramid Pooling Fast (SPPF) module, aimed at improving the sensitivity and discriminative capability of the YOLOv11 detection network toward minute lesion areas.

The TSFA module comprises three structurally complementary attention branches, each designed to enhance input features at the spatial, channel, and neuron levels, respectively. This configuration constitutes a lightweight yet expressive triple-attention mechanism. Specifically, the spatial attention branch is derived from the Depthwise Convolution component of the ACmix module [38], which models each channel independently in the spatial domain without increasing parameter complexity. This design effectively preserves local texture details and edge variations, making it particularly suitable for detecting small, localized lesions or blurred boundaries on fish skin. The channel attention branch adopts the Efficient Channel Attention (ECA) mechanism [39], which eliminates the dimensionality reduction process inherent in traditional Squeeze-and-Excitation (SE) modules. Instead, it employs a one-dimensional convolution to model channel-wise dependencies based on globally pooled descriptors. This approach establishes localized inter-channel relationships and significantly enhances the response to low-activation yet diagnostically critical channels, thereby improving feature discrimination in disease detection. The neuron-level attention branch is based on the SimAM module [40], which introduces a closed-form energy function to evaluate the relative importance of each individual pixel within the overall feature map distribution. By comparing each neuron’s activation to the global mean and variance, SimAM generates a discriminative spatial weight map without the need for additional trainable parameters. This enables selective amplification of key pixel regions within the original feature map, while effectively suppressing irrelevant background information.

**Figure 6:**
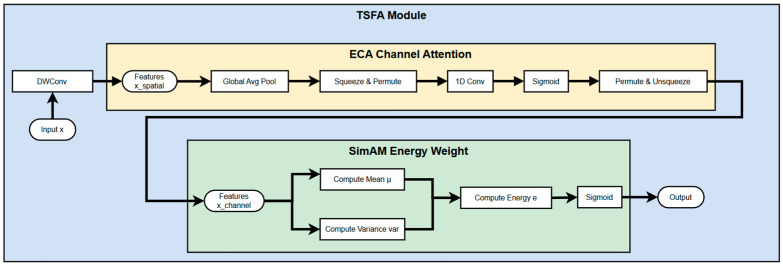
TSFA Module Structure Diagram.

As illustrated in Figure 6, the TSFA module’s data flow is delineated into three distinct pathways: a spatial perception path, a channel enhancement path, and an energy-weighted path. This architecture underscores the module’s advantages in fine-grained object detection tasks, particularly in scenarios where precise localization of subtle or low-contrast pathological features is critical.

The traditional SPPF (Spatial Pyramid Pooling Fast) module constructs multi-scale receptive field features of local regions through successive MaxPooling operations, effectively expanding the model’s spatial perception range. However, it lacks an attention mechanism that emphasizes salient regions after feature fusion, particularly underperforming in the detailed representation of lesions. To address this limitation, this study proposes the integration of a Triple-Stage Feature Attention (TSFA) module following the concatenation of SPPF output features, thereby enhancing the representation of key regional features. This module is applicable to various lightweight-object detection tasks, and is especially effective in improving detection robustness and detail sensitivity for small targets in fish-disease recognition scenarios.

**Figure 7:**
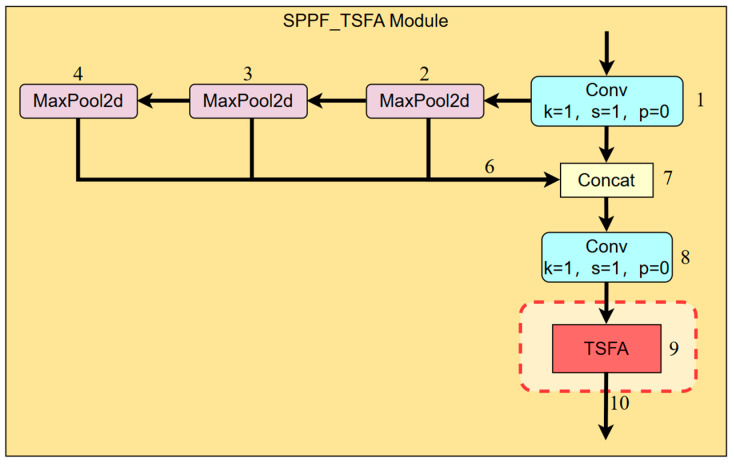
SPPF_TSFA Module Structure Diagram.

Specifically, the input features are first channel-compressed via a 1 × 1 convolution before entering the SPPF module. The module sequentially applies three sets of MaxPooling operations with shared kernel sizes to extract multi-scale spatial information. Subsequently, the original features are concatenated with the pooled outputs and passed through a 1 × 1 convolution to restore the channel dimension. The resultant feature map is then fed into the TSFA module, which performs three sequential operations: spatial modeling via Depthwise convolution, channel enhancement through ECA convolution, and energy weighting employing the SimAM mechanism. The final output features combine global contextual awareness with local lesion sensitivity, significantly improving the model’s localization precision and classification reliability for small-lesion areas in fish-disease detection. The architecture of the SPPF_TSFA module is illustrated in Figure 7.

#### 2.3.3. Optimization of Local Feature Extraction Mechanism by Incorporating Windmill-like Convolution

In fish-disease image recognition tasks, the substantial degradation of image quality—manifested notably by uneven illumination, strong background interference, and blurred target textures—significantly hampers the accurate extraction of lesion region features by models, thereby increasing the risk of misclassification. To address these challenges, this study proposes the integration of the Pinwheel-shaped Convolution (PConv) module [41] into the ShuffleBlock architecture [42], replacing the conventional 1 × 1 convolutional layers. This modification aims to markedly enhance the spatial awareness and target responsiveness of low-level feature extraction, ultimately improving the detection performance of fish-disease recognition systems under complex conditions.

The design of PConv (Polarized Convolution) is inspired by its asymmetric kernel structure, which arranges convolutional weights in a pinwheel-like pattern. This architecture effectively enlarges the receptive field, while maintaining low computational complexity. In contrast to conventional 1 × 1 convolutions, which have a receptive field limited to a single pixel, PConv introduces a spatially asymmetric sampling strategy within the kernel (e.g., selectively activating weights in a 3 × 3 kernel in a pinwheel configuration), thereby enabling the extraction of broader spatial contextual information while preserving fine-grained local texture details. The theoretical advantages of PConv can be summarized as follows: (1) the asymmetric kernel design enhances directional sensitivity to lesion edges and textures, making it particularly well-suited for capturing the linear filamentous structures of Saprolegnia (water mold) infections or the irregular margins of ulcerative lesions; (2) the sparse kernel configuration reduces the parameter count by approximately 22.2%, thereby decreasing computational overhead; and (3) the receptive field is expanded by about 178%, improving the model’s ability to distinguish local targets in scenarios involving overlapping fish. Conceptually, the pinwheel-shaped kernel of PConv is tailored to model the spatial heterogeneity of fish-disease manifestations (e.g., radially distributed fungal hyphae or non-uniform ulcerative textures), thereby enabling more effective extraction of complex pathological features.

**Figure 8:**
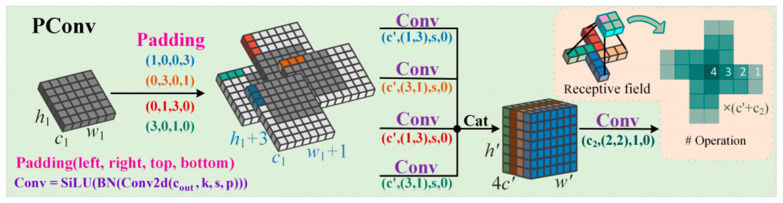
PConv Module Schematic Diagram: The “#” in the figure caption is used to mark specific steps in the figure.

In underwater environments, fish lesions often present with low contrast and blurred boundaries, due to factors such as water flow disturbances, uneven lighting, and background impurities. Traditional convolutional kernels (e.g., standard 1 × 1 or 3 × 3 kernels) struggle to distinguish lesions from background noise under such conditions, and may lead to target confusion, especially in overlapping multi-fish scenarios. The asymmetric kernel of PConv allows for dynamic adjustment of feature responses across different directions, thereby enhancing sensitivity to lesion edge textures and enabling more precise localization of diseased regions in visually complex backgrounds. For instance, in the case of Saprolegnia-induced cotton-like hyphae, PConv can effectively capture the linear distribution along the fish body surface; for ulcerative syndrome, it models the local spatial structure of irregular lesions by leveraging its expanded receptive field. This synergy of “receptive field expansion” and “parameter reduction” makes PConv particularly advantageous for lightweight neural network architectures deployed in resource-constrained environments. A schematic illustration of the PConv architecture is provided in Figure 8.

The ShuffleNet V2 architecture, owing to its efficient channel shuffle mechanism and branched structural design, demonstrates a favorable trade-off between computational cost and performance in mobile image recognition tasks. Its fundamental building block, the ShuffleBlock, comprises two parallel branches: a shortcut (identity) path and a learnable path. The learnable path incorporates two sequential 1 × 1 pointwise convolutions responsible for channel compression and feature transformation, while cross-channel information fusion is achieved through element-wise addition followed by channel shuffle operations.

**Figure 9:**
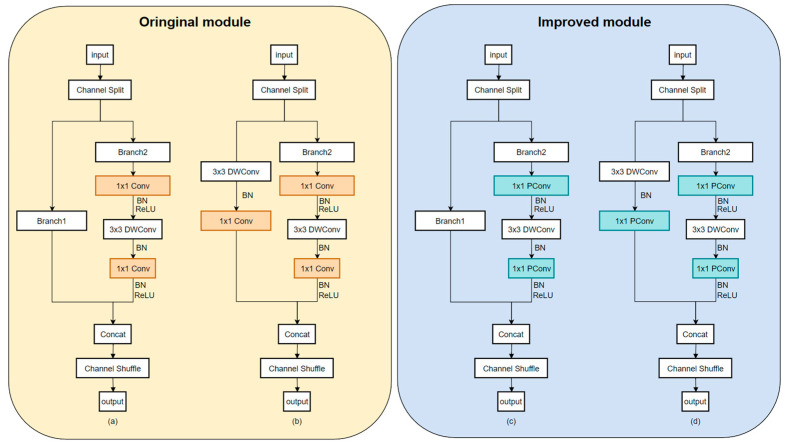
Basic units of ShuffleBlocks: (**a**) basic feature extraction structure, (**b**) ShuffleBlock structure for downsampling, (**c**) improved feature extraction structure, (**d**) improved downsampling structure.

Leveraging the architectural characteristics of ShuffleNet V2, this study proposes a structural modification to the 1 × 1 convolutions within the channel branch. In the original ShuffleBlock, the input is split into two branches: one remains unchanged, while the other sequentially passes through a 1 × 1 convolution, a 3 × 3 depthwise separable convolution, and a subsequent 1 × 1 convolution, to accomplish feature reconstruction and channel integration. The proposed enhancement replaces both 1 × 1 convolutions in this branch with PConv modules (kernel size k = 3), aiming to improve the model’s capacity for capturing local spatial structures. The overall design of this module is illustrated in Figure 9, where Figure 9a,b depict the original ShuffleBlock architecture, and Figure 9c,d show the improved version with 1 × 1 convolutions substituted by PConv.

By establishing a stronger coupling between spatial information awareness and channel representation through the PConv module, alongside its direction selectivity and local feature enhancement capabilities, the method enriches the diversity of expressions across different channels. This not only enhances the efficiency of information interaction and discriminative power, but also improves the modeling of complex fish-disease image features without significantly increasing computational overhead.

In summary, the strategy proposed in this study, which replaces the 1 × 1 convolution in the ShuffleBlock with PConv, constitutes a lightweight and effective modular structural-optimization approach. By enhancing the modeling capacity of the underlying feature space, this method effectively mitigates the adverse effects of uneven illumination and complex backgrounds on disease recognition models. It demonstrates strong structural generalizability and practical applicability, providing a valuable reference for network architecture optimization in real-world scenarios such as underwater disease diagnosis, under challenging imaging conditions.

#### 2.3.4. Scale-Aware Dynamic Loss Mechanism for Efficient Extraction of Dynamic Features

The external manifestations of fish diseases exhibit significant scale and morphological variability across different stages of disease progression. For example, early-stage Saprolegniasis typically presents as small, white hyphal spots, whereas in later stages it may develop into extensive cotton-like patches. Conventional loss functions such as the Complete IoU (CIoU) often struggle to accurately localize small-lesion targets, and are prone to high false-detection rates, particularly in scenarios involving blurry lesion boundaries and substantial variation in lesion morphology and size. To address these challenges, this study introduces the Scale-based Dynamic IoU Loss (SDIoU) [41] into the context of fish-disease detection in aquaculture. SDIoU dynamically adjusts the weighting between scale loss and localization loss, thereby enhancing the model’s adaptability to lesion scale and shape variations corresponding to different disease stages. This leads to improved detection accuracy while reducing both false negatives and false positives. The key contribution of this study lies in validating the effectiveness of SDIoU under complex aquaculture conditions, such as uneven lighting, overlapping fish, and small-lesion targets, and in optimizing its implementation specifically for fish-disease detection tasks.

It is important to clarify that the SDIoU loss function is designed based on static image datasets, and aims to enhance robustness to static variations in lesion scale and morphology. It does not attempt to model the temporal progression of diseases, and thus is not intended for dynamic disease tracking over time.

To address the issue of instability in the Intersection over Union (IoU) of symptomatic regions, SDIoU incorporates a dynamic weight-adjustment mechanism. Specifically, it modulates the relative contributions of scale loss and localization loss based on the size of the target region. For bounding box (BBox) annotations, SDIoU first defines a base loss function, wherein scale loss and localization loss are formulated separately:

(1) $$L_{BS}=1-IoU+\alpha v,\;L_{BL}=\frac{\rho^{2}(b_{p},b_{gt})}{c^{2}}$$

As shown in Equation (1), IoU represents the Intersection over Union (IoU) between the predicted bounding box and the ground-truth bounding box, αv quantifies the consistency of the bounding boxes’ aspect ratios, and ρ corresponds to the Euclidean distance. $b_{p}$ and $b_{gt}$ denote the center points of the predicted and ground-truth bounding boxes, respectively, while c indicates the length of the diagonal of the bounding boxes. This formulation ensures that the optimization process simultaneously considers both the overlap region and the relative positions of the bounding-box centers. However, the scale variations in fish-disease symptoms challenge the adequacy of a fixed-loss weighting scheme to effectively accommodate all scenarios. To address this, the SDIoU loss introduces a scale factor $\beta_{B}$, which dynamically modulates the weights of $L_{BS}$ and $L_{BL}$, thereby enhancing adaptability across different scales:

(2) $$\beta_{B}=\min\left(\frac{B_{gt}}{B_{gt\;max}}\times R_{OC}\times \delta ,\;\delta \right)$$

As shown in Equation (2), $B_{gt}$ denotes the area of the ground-truth bounding box, $B_{gt\;max}$ = 81 represents the maximum target scale, $R_{OC}$ corresponds to the ratio between the original image size and the current feature map size, and δ is a tunable parameter. This formulation restricts the weight range through scale normalization, thereby ensuring the stability of detection for small-scale symptoms. The final SDIoU loss function (SDB) incorporates these dynamic weights, and is formally defined as presented in Equations (3) and (4).

(3) $$\beta_{L_{BS}}=1-\delta +\beta_{B},\;\beta_{L_{BL}}=1+\delta -\beta_{B}$$

(4) $$L_{SDB}=\beta_{L_{BS}}\times L_{BS}+\beta_{L_{BL}}\times L_{BL}$$

**Figure 10:**
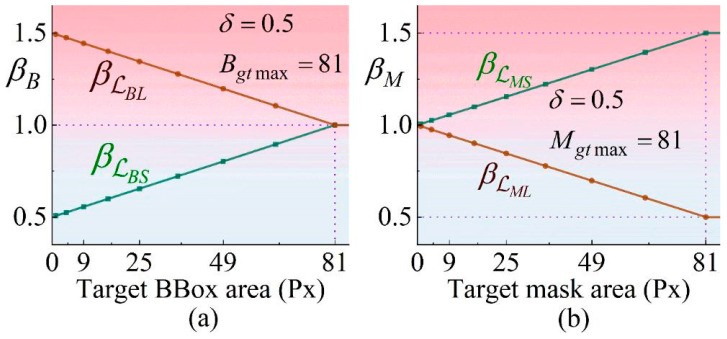
(**a**) The values of the weights $\beta_{B}$ in Sloss and Lloss related to the BBox area. (**b**) The values of the weights $\beta_{M}$ in Sloss and Lloss related to the target mask area.

For bounding-box (BBox) annotations, the attention weight assigned to the scale loss (Sloss) tends to be relatively low when the target size is small, as illustrated in Figure 10a. In contrast, mask annotations demonstrate superior performance in handling small-scale or irregularly shaped objects, thereby further enhancing detection accuracy. Moreover, the location loss (Lloss) associated with mask annotations is computed based on the average position of all objects within the image. Consequently, when an object is missed during detection, this loss component struggles to converge effectively, which may increase the risk of false positives. To address this issue and strengthen the model’s focus on critical regions under mask supervision, it is necessary to elevate the relative weight of Sloss within the overall loss function. This adjustment aims to improve the model’s discriminative capability in scenarios involving complex object structures and weak edge conditions, as depicted in Figure 10b.

Furthermore, the prevalent presence of blurred boundaries in fish-disease images exacerbates the volatility of the Intersection over Union (IoU) metric, thereby compromising the stability of the model. To address this issue, the SDIoU metric introduced in this study draws inspiration from the SLS loss function. Specifically, SDIoU is architected by decomposing the overall loss into two components: the mask scale loss (L_MS) and the positional loss (L_ML). Moreover, it dynamically adjusts the weighting of these loss components according to the scale of the target, enabling a more stable and adaptive training process.

(5) $$L_{MS}=1-\omega \frac{\left|M_{p}\cap M_{gt}\right|}{\left|M_{p}\cup M_{gt}\right|}\;$$

(6) $$\beta_{M}=\min\left(\frac{M_{gt}}{M_{gt\;max}}\times R_{OC}\times \delta ,\;\delta \right)$$

(7) $$L_{ML}=1-\frac{\min(d_{p},d_{gt})}{\max(d_{p},d_{gt})}+\frac{4}{\pi^{2}}(\theta_{p}-\theta_{gt}{)}^{2}$$

As illustrated in Equations (5)–(7), $M_{p}\;and\;M_{gt}$ correspond to the predicted and ground-truth mask pixel collections, respectively; $\beta_{M}$ denotes the mask influence coefficient; $d_{p}\;and\;d_{gt}$ represent the polar coordinate distances of the mean predicted and ground-truth pixels from the origin; $\theta_{p}\;and\;\theta_{gt}$ correspond to the polar coordinate angles of the pixel means; and ω characterizes the mask discrepancy. This mechanism, by emphasizing scale-aware loss on small-scale lesions, effectively mitigates the false-positive rate caused by ambiguous boundaries. 

These findings demonstrate that SDIoU, by dynamically adjusting the weights of scale and localization losses, significantly enhances the deep learning model’s ability to adapt to variations in lesion size and morphology on fish bodies. This approach offers an efficient and robust optimization strategy for disease detection models. Moreover, its simple yet effective design is not only applicable to fish-disease recognition, but can also be generalized to other small-object detection tasks. Future research could further validate the generalizability of this method across a wider range of target types and explore more sophisticated approaches for computing the dynamic weighting factors, to improve performance.

## 3. Experiment and Analysis

### 3.1. Experimental Environment Configuration

The model training and evaluation conducted in this study were performed on a computational platform configured as follows: a central processing unit (CPU) of AMD Ryzen 7 7840 H integrated with a Radeon 780 M graphics unit, accompanied by 16 GB of physical memory. The system employed an NVIDIA GeForce RTX 4060 graphics processing unit (GPU) with 8 GB of dedicated video memory. The operating system was Microsoft Windows 11. The deep-learning development environment utilized for the experiments was based on Python 3.9 and implemented on the PyTorch 2.2.2 framework, leveraging CUDA version 12.2 to facilitate GPU-accelerated computation. To minimize the influence of stochastic variability inherent in deep learning models, the following measures were implemented: (1) a fixed random seed (seed = 42) was used to ensure the reproducibility of the initial experiments; and (2) each model was independently trained three times, using different random seeds to evaluate the stability and statistical significance of model performance. The training parameters for all models are detailed in Table 5.

### 3.2. Model Training Process

In this study, the model was trained for a total of 100 epochs, employing a batch size of 16 to iteratively update the parameters. The initial learning rate was set to 0.01 to regulate the magnitude of gradient updates during the early training stages, thereby ensuring efficient convergence while preventing excessive weight oscillations.

**Figure 11:**
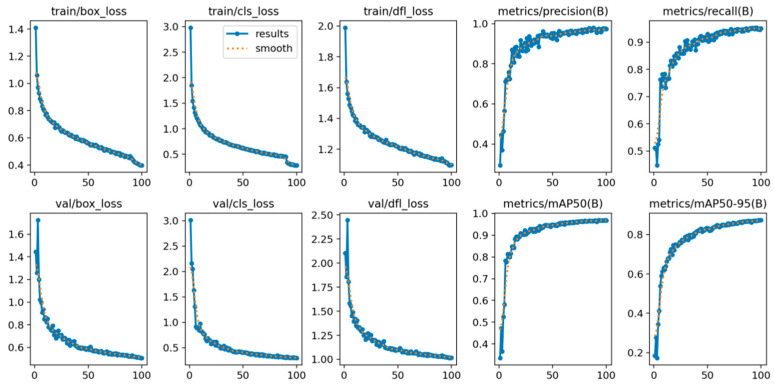
Model training and validation metrics.

The training curves are presented in Figure 11, which systematically illustrates the dynamic evolution of the training process for the improved YOLOv11 model applied to fish-disease detection. The figure comprises nine subplots depicting the trends over epochs for both the training (train) and validation (val) datasets. These subplots include bounding-box loss (box loss), classification loss (cls loss), and distribution focal loss (dfl loss), as well as key performance metrics: Precision, Recall, and mean average Precision (mAP). The convergence behavior of the loss functions during training and validation indicates that train/box_loss and val/box_loss started at approximately 1.4 and ultimately converged below 0.4; train/cls_loss and val/cls_loss decreased progressively from initial values of 2.5 and 2.0 to below 0.5 and 1.0, respectively; similarly, train/dfl_loss and val/dfl_loss stabilized around 1.2 and 1.25, descending from initial values of 1.8 and 1.75.

These outcomes demonstrate that the PC_Shuffleblock module, enhanced by pinwheel-shaped convolutions (PConv), effectively improves spatial awareness, while the SPPF_TSFA module facilitates robust multi-scale feature fusion combined with a triple-attention mechanism. This synergy enables efficient extraction of complex fish-disease features, significantly enhancing the model’s robustness in object localization, classification, and distribution modeling. Moreover, the consistent loss trends observed between the validation and training sets further substantiate the model’s generalization capability.

At the level of performance evaluation metrics, the curves of metrics/Precision(B) and metrics/Recall(B) exhibited a pronounced upward trend, increasing from initial values of 0.6 and 0.5 to approximately 0.98 and 0.96, respectively. This highlights the model’s superior capability in identifying positive samples and controlling missed detections. Additionally, metrics/mAP50(B) and metrics/mAP50-95(B) improved to nearly 0.98 and 0.88, respectively, indicating excellent detection accuracy across varying IoU thresholds, with particularly stable performance maintained under stringent IoU requirements. These outcomes are attributed to the incorporation of the SDIoU loss function, which introduces a scale dynamic-adjustment mechanism. By adaptively modulating the weights of scale and localization losses, this mechanism effectively mitigates IoU fluctuations in small targets, thereby enhancing the model’s sensitivity to early-stage, subtle lesions in fish diseases. 

In summary, the comprehensive experimental results robustly validate the superior performance of the improved YOLOv11 model in fish-disease detection tasks. The synergistic optimization of multiple modules significantly advances both detection accuracy and generalization ability, providing a solid technical foundation for intelligent diagnosis of fish diseases in complex aquaculture environments. 

### 3.3. Model Evaluation Metrics

To comprehensively evaluate the proposed object detection model in terms of lightweight design, detection accuracy, and inference efficiency, multiple evaluation metrics are introduced from various perspectives. For assessing model compactness, the number of parameters (Parameters), floating-point operations (FLOPs), and model sizes (Model Sizes) are employed to quantify the computational and storage overhead. Regarding accuracy, Precision (P), Recall (R), and mean average precision (mAP) are selected as the primary metrics, with mAP calculated at an Intersection over Union (IoU) threshold of 0.5 (mAP_0.5_) serving as the key accuracy indicator. The detailed computational formulas are presented in Equations (8)–(11). To account for model stochasticity, all evaluation metrics were reported as mean ± standard deviation across multiple experimental runs. A paired *t*-test was conducted to assess whether the performance difference in mAP_0.5_ between the proposed YOLO-TPS model and the baseline YOLOv11n model was statistically significant.

(8) $$P=\frac{T_{P}}{T_{P}+F_{P}}\times 100\%$$

(9) $$R=\frac{T_{P}}{T_{P}+F_{N}}\times 100\%$$

(10) $$AP=\int_{0}^{1}P\cdot dR$$

(11) $$mAP=\frac{1}{N}\sum_{i=1}^{N}{AP}_{i}$$

In the aforementioned evaluation metrics, $T_{P}$ (True Positive) denotes the number of positive samples correctly identified by the model; $F_{N}$ (False Negative) refers to the number of positive samples that the model failed to detect; and $F_{P}$ (False Positive) indicates the number of negative samples incorrectly classified as positive by the model. Furthermore, *N* represents the total number of categories involved in the evaluation [43]. In this study, *N* is set to 6, corresponding to 6 types of fish diseases.

### 3.4. Experimental Results

#### 3.4.1. PC_Shuffleblock Module Insertion-Position Test Analysis

To systematically evaluate the impact of integrating the PC_Shuffleblock module at different hierarchical levels within the network architecture on fish-disease detection performance, this study designed five comparative experimental groups. Specifically, the PC_Shuffleblock module was inserted at various network depths: PC_Shuffleblock (P0/P1), Shuffleblock (P7/P17/P20), PC_Shuffleblock (P5/P7), PC_Shuffleblock (P3/P5), and PC_Shuffleblock (P17/P20). All experiments were conducted under a unified baseline model, dataset, training parameters, and evaluation metrics. Each model was trained for 100 epochs with a batch size of 16, employing the SGD optimizer with an initial learning rate of 0.01. The evaluation metrics included mean Average Precision at IoU 0.5 (mAP_0.5_), Precision, and Recall, to comprehensively assess the model’s detection accuracy and robustness. As shown in Table 6, the PC_Shuffleblock (P17/P20) configuration achieved the best performance, with an mAP_0.5_ of 97.2%, Precision of 97.9%, and Recall of 95.1%, significantly outperforming the other groups. These results indicate that embedding the PC_Shuffleblock module at deeper network layers (layers 17 and 20) effectively enhances the spatial modeling capacity of deep features through the asymmetric receptive field design of the PConv (pinwheel convolution). This enhancement facilitates superior extraction and recognition of complex fish-disease characteristics.

#### 3.4.2. Ablation Experiment

To systematically evaluate the individual and synergistic effects of each improved module in the fish-disease detection task, eight groups of ablation experiments were designed based on the YOLOv11n baseline network. All experiments were conducted under identical training environments and parameter settings. The results are presented in Table 7.

The experimental outcomes demonstrate that the introduction of the PC_Shuffleblock module significantly enhances the detection performance of the YOLOv11n model, with mAP_0.5_ increasing from 93.4% to 95.1%, Precision improving from 92.5% to 95.1%, and Recall rising from 90.4% to 92.8%. This improvement indicates that the PC_Shuffleblock module, leveraging the flexible receptive field design of its pinwheel-shaped convolution (PConv), effectively strengthens the model’s ability to capture edge details and complex textures at the feature extraction stage, thereby improving detection accuracy and robustness.

Moreover, the integration of the SPPF_TSFA module also yields performance gains. Although mAP_0.5_ shows a modest increase, from 93.4% to 94.3%, Precision and Recall are elevated to 93.8% and 90.7%, respectively. This suggests that the synergistic effect of multi-scale feature-fusion and spatial-attention mechanisms in the SPPF_TSFA module effectively enhances the model’s contextual modeling capability and its ability to detect small targets.

Furthermore, the replacement of the loss function with SDIoU loss optimizes the model’s gradient responsiveness to samples of varying quality. The scale-dynamic regulation mechanism alleviates training perturbations caused by the instability of small-object IoU, resulting in an mAP_0.5_ increase to 94.7%, with Precision and Recall improving to 94.2% and 91.7%, respectively. Consequently, the model exhibits enhanced generalization performance under complex backgrounds and varying target scales.

These results comprehensively validate the complementarity among the modules in terms of structural design and optimization objectives, further demonstrating that the multi-module collaborative integration strategy significantly enhances the overall performance of the model in real-world detection tasks.

#### 3.4.3. Contrast Experiment

To validate the performance advantages of the proposed YOLO-TPS model in fish-disease detection tasks, a systematic comparative analysis was conducted against multiple mainstream object-detection models, including Faster R-CNN, SSD, YOLOv5, YOLOv8n, YOLOv9n, YOLOv10n, and YOLOv11n. The experimental results, summarized in Table 8, present the performance metrics of each model on the test dataset, reported as the mean and standard deviation over three independent training runs. The YOLO-TPS model comprises 2.513 million parameters, significantly fewer than Faster R-CNN (44.297 million) and SSD (30.245 million). Its floating-point operations (FLOPs) amount to 6.9 G, demonstrating markedly lower computational complexity compared to Faster R-CNN (207 G) and SSD (37.8 G). The model size is 5.4 MB, which is substantially smaller than that of Faster R-CNN (315 MB) and SSD (104 MB). Although the YOLO-TPS model’s parameter count and FLOPs are marginally higher than those of YOLOv11n (2.583 million parameters, 6.3 G FLOPs), and its model size (5.4 MB) is slightly larger than YOLOv11n’s (5.2 MB), it exhibits competitive advantages in key performance metrics, reflecting a balanced trade-off between lightweight design and detection efficiency.

Regarding performance evaluation, the YOLO-TPS model achieves a mean average Precision at IoU 0.5 (mAP_0.5_) of 97.2%, significantly outperforming Faster R-CNN (61.2%), SSD (56.2%), YOLOv5 (90.2%), YOLOv8n (91.4%), YOLOv9n (92.3%), YOLOv10n (92.2%), and YOLOv11n (93.4%), with respective improvements of 36.0%, 41.0%, 7.0%, 5.8%, 4.9%, 5.0%, and 3.8%. In terms of Precision, YOLO-TPS attains 97.9%, exceeding Faster R-CNN (70.5%), SSD (53.1%), YOLOv5 (87.4%), YOLOv8n (91.6%), YOLOv9n (88.8%), YOLOv10n (91.5%), and YOLOv11n (92.5%) by 27.4%, 44.8%, 10.5%, 6.3%, 9.1%, 6.4%, and 5.4%, respectively. The Recall rate of YOLO-TPS reaches 95.1%, also surpassing Faster R-CNN (65.9%), SSD (44.2%), YOLOv5 (86.9%), YOLOv8n (90.3%), YOLOv9n (89.0%), YOLOv10n (89.1%), and YOLOv11n (90.4%) by 29.2%, 50.9%, 8.2%, 4.8%, 6.1%, 6.0%, and 4.7%, respectively. Although YOLOv11n demonstrates comparable performance on certain metrics, the superior mAP_0.5_ score of YOLO-TPS (3.8% higher), combined with its overall Precision and Recall performance, underscores its outstanding capability in fish-disease detection.

The standard-deviation results indicate that YOLO-TPS demonstrated superior stability across multiple runs, with a standard deviation of 0.3% in mAP_0.5_, which is lower than that of YOLOv11n (0.4%), reflecting the enhanced robustness of the proposed model. To assess the statistical significance of the observed performance improvement, a paired *t*-test was conducted, comparing the mAP_0.5_ values of YOLO-TPS and YOLOv11n, based on test results from three independent runs. The results revealed that YOLO-TPS achieved a significantly higher mAP_0.5_ than YOLOv11n (*p* = 0.002, *p* < 0.01), confirming that the performance gain is not attributable to random variation, but is instead driven by the architectural enhancements incorporated in the model design.

In summary, the YOLO-TPS model excels in terms of parameter count (2.513 million), FLOPs (6.9 G), model size (5.4 MB), and performance metrics (mAP_0.5_ 97.2%, Precision 97.9%, Recall 95.1%), demonstrating significant advantages over traditional and contemporary YOLO-based models. These results substantiate the efficiency and robustness of YOLO-TPS in fish-disease detection tasks, providing important theoretical insights and practical references for the advancement of intelligent diagnostic technologies in aquaculture.

**Figure 12:**
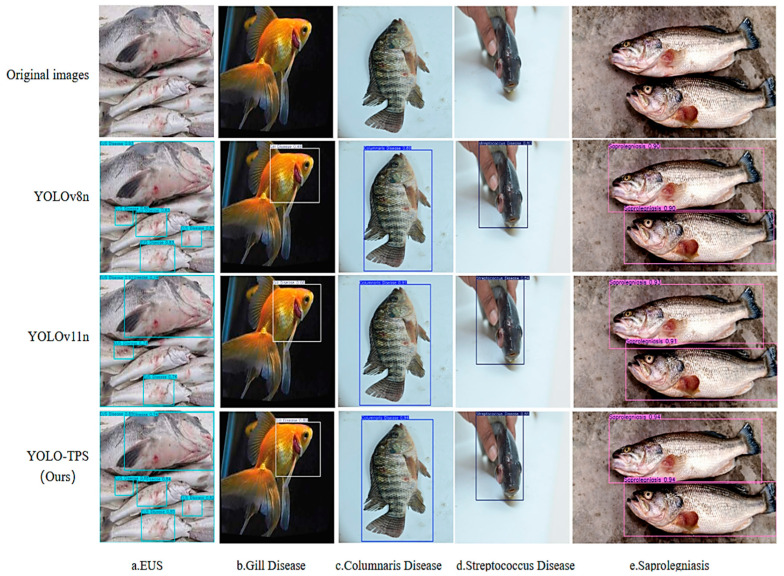
Comparison of model detection results: the first row displays original images: (**a**) multi-fish overlap with dense fish bodies, (**b**,**c**) single clear fish, highlighting lesion details, (**d**) complex background with hand- and ground-reflection interference, and (**e**) multi-fish alignment with parallel fish bodies. The second-to-fourth rows show detection results of YOLOv8n, YOLOv11n, and YOLO-TPS, respectively, with bounding boxes in different colors, annotated with confidence scores and pathology labels.

We compared the predictions of the three models with the best combined performance in the results of the comparison experiments, and the confidence level was set to 0.5. Figure 12 demonstrates the results of YOLOv11, YOLOv8n, and YOLO-TPS for the detection of different fish diseases.

The experiment selected images representing four typical scenarios, including complex cases such as overlapping multiple fish, single clear fish, interference from complex backgrounds, and multiple fish arranged side by side. The detection results are presented in Figure 12. The first row of the figure displays the original images: the first column depicts a densely stacked scene of multiple fish on the water surface; the second and third columns show images of individual fish; the fourth column presents a scenario with a single fish held by hand, where the background includes hand- and ground-reflection interference; the fifth column illustrates multiple fish aligned side by side. The subsequent three rows show detection results from YOLOv8n, YOLOv11n, and YOLO-TPS, respectively. Detection bounding boxes are color-coded and annotated with confidence scores and pathological-category labels.

In the overlapping multiple-fish scenario, YOLOv8n and YOLOv11n identified some fish but exhibited notable bounding-box deviations, with several targets either mislocalized or missed, indicating incomplete detection. In contrast, YOLO-TPS demonstrated superior robustness in dense target detection by accurately localizing bounding boxes that fully encompassed all fish, and correctly labeling pathological categories.

In the single-fish detection scenario, both YOLOv8n and YOLOv11n successfully localized the targets; however, their bounding boxes showed lower tightness and confidence scores, and were more susceptible to background interference. Conversely, YOLO-TPS produced bounding boxes that closely conformed to the fish contours, achieved higher confidence scores, and effectively suppressing background noise while accurately annotating pathological features.

In the multiple-fish side-by-side scenario, YOLO-TPS further showcased its advantages, generating bounding boxes that precisely adhered to each fish’s contour, with confidence scores exceeding those of YOLOv8n and YOLOv11n. These improvements can be attributed to YOLO-TPS’s integration of the PC_Shuffleblock module, which enhances spatial modeling capability; the SPPF_TSFA module, which extracts multi-scale features for small lesions; and the dynamically optimized SDIoU loss function. Together, these components contribute to the model’s outstanding performance in detection accuracy, bounding-box precision, and prediction reliability, thereby providing an efficient and robust technical solution for intelligent diagnosis in complex fish-disease detection scenarios.

To further evaluate YOLO-TPS’s detection performance across different fish-disease types, this study conducted a statistical analysis of Precision, Recall, and mean Average Precision at IoU 0.5 (mAP_0.5_) for six fish-disease categories plus a healthy class, with results summarized in Table 9. Although certain categories, such as EUS, exhibited relatively lower Recall and mAP values—highlighting ongoing challenges in detecting complex lesions—YOLO-TPS demonstrated excellent average detection accuracy across other categories. Overall, the model achieved a Precision of 97.9%, Recall of 95.1%, and mAP_0.5_ of 97.2%, reflecting strong generalization ability and robustness in multi-class fish-disease detection tasks.

#### 3.4.4. Comparison of Model Feature Visualization

**Figure 13:**
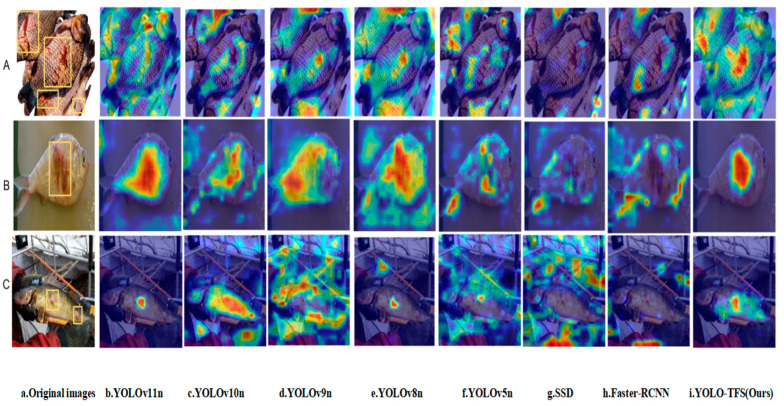
Comparison of Model Heatmap Results: (**A**) multi-fish overlap, (**B**) single clear fish, and (**C**) complex background interference. Yellow bounding boxes in original images indicate lesion areas.

Gradient-weighted Class Activation Mapping (Grad-CAM) [44] is a visualization technique designed to elucidate the spatial regions within input images that deep learning models prioritize during image classification tasks. By leveraging the gradient information of the model, Grad-CAM precisely identifies the input features that are critical for predicting specific classes. In this study, heatmaps were generated using a confidence threshold of 0.65 to quantify the model’s response intensity across different regions, where higher color brightness in the heatmap corresponds to greater influence on the prediction outcome. The experiments encompassed three scenarios: Scenario A, representing overlapping multiple-fish conditions, Scenario B, depicting a single fish in a clear setting, and Scenario C, involving scenes with complex background interference. The original images feature yellow bounding boxes indicating lesion areas annotated by the Fish Disease Research Institute. The heatmap results, illustrated in Figure 13, provide intuitive and direct evidence for evaluating the model’s performance.

As illustrated in Figure 13, the improved YOLO-TPS model demonstrates superior performance in identifying fish-disease characteristics. Compared to other models—including YOLOv11n, YOLOv10n, YOLOv9n, YOLOv8n, YOLOv5, SSD, and Faster-RCNN—YOLO-TPS exhibits outstanding adaptability and robustness across diverse scenarios. In cases of fish overlap, the model precisely focuses on pathological lesions, effectively mitigating misclassification caused by background interference. Under clear single-fish conditions, the model accurately localizes disease features, despite the presence of edge noise. Furthermore, in complex backgrounds with substantial interference, YOLO-TPS successfully suppresses noise and reliably detects pathological regions. These findings indicate that the enhanced model generates more concentrated and targeted heatmap responses, reflecting heightened sensitivity to critical fish-disease features and enabling better capture of subtle lesion variations. This advancement is significant for improving diagnostic accuracy in aquaculture disease detection, particularly in high-precision applications under complex environmental conditions, thereby underscoring the model’s practical value.

## 4. Discussion

In this study, we propose an enhanced detection model, YOLO-TPS, based on YOLOv11n, which demonstrates significant performance improvements in fish-disease image recognition tasks. By incorporating a multi-module collaborative optimization framework—comprising the SPPF_TSFA module, the PC_Shuffleblock module, and the Scale-Dynamic IoU (SDIoU) loss function—the model exhibits superior accuracy and robustness in handling complex backgrounds, detecting small targets, and dynamically modeling disease-feature variations.

### 4.1. Effectiveness Analysis of the Tri-Level Selective Focus Attention

To address the challenge of effectively extracting subtle lesion features in fish-disease images, this study proposes an SPPF_TSFA module integrated with a triple-attention mechanism within the backbone of YOLOv11. This module enhances feature responses across spatial, channel, and neuron levels. Specifically, it incorporates the spatial awareness capabilities of ACmix [38], the local channel modeling strength of ECA [39], and the parameter-free neuron weighting strategy of SimAM [40], collectively improving the model’s sensitivity in detecting small targets indicative of early fish-disease symptoms, such as initial ulcers and microbial adhesion. This approach aligns with the successful application of multi-scale fusion and attention mechanisms in agricultural disease identification reported by Qian et al. [3], Stephen et al. [7], and Dulal et al. [17], thereby validating the efficacy of attention mechanisms in accurately localizing critical lesions within complex image backgrounds.

### 4.2. Lightweight Architecture and Adaptability to Complex Backgrounds

To address common challenges in aquaculture environments, such as uneven illumination, background interference, and target occlusion, this study introduces the PC_Shuffleblock module into the detection head. This module is designed based on pinwheel-shaped convolution (PConv), and employs an asymmetric sparse-connection strategy to significantly enlarge the effective receptive field while reducing computational redundancy [41]. By embedding the ShuffleBlock architecture, the module not only enhances spatial modeling capability, but also maintains a lightweight profile, making it well-suited for deployment on edge devices. Similar structural designs have been validated in applications like object detection and crop recognition; for instance, Gao et al. [6] achieved high-precision detection of maize diseases using lightweight convolutions, while Bhuyan et al. [13] improved edge response in tea-leaf disease detection through the CBAM module. Experimental results in this study further demonstrate that PC_Shuffleblock exhibits strong adaptability to challenges such as edge blurring and texture degradation, thereby significantly improving detection accuracy.

### 4.3. Necessity of Scale-Adaptive Loss Functions

In response to the pronounced scale dynamics exhibited by the external manifestations of fish diseases throughout disease progression, traditional static loss functions such as CIoU and DIoU are inadequate for addressing the significant fluctuations in Intersection over Union (IoU) encountered during model training. To overcome this limitation, this study proposes a Scale-Dynamic IoU (SDIoU) loss function, which incorporates a dynamic weighting factor based on the target size to modulate the contributions of positional and scale errors to the overall loss. This approach effectively mitigates detection instability caused by small-object misclassification and boundary ambiguity. Furthermore, this mechanism aligns with the perspective emphasized by Mujahid et al. [14], who advocate for loss function designs that account for target scale sensitivity, underscoring the critical role of loss function optimization in high-precision tasks such as disease detection.

### 4.4. Failure-Case Analysis of Model

To provide a comprehensive assessment of the YOLO-TPS model’s performance, this study conducted a focused analysis of failure cases in detecting extremely small lesions, thereby identifying the model’s limitations and potential areas for improvement in such scenarios. The majority of failure cases were associated with lesions occupying less than 0.5% of the image area, as exemplified by the minute parasitic lesion shown in Figure 13C.

In Figure 13C, the YOLO-TPS model failed to detect the minute parasitic lesion, resulting in a missed detection (confidence score < 0.65). Two primary factors contributed to this failure: (1) the lesion area was exceedingly small (<0.5% of the image area), and at the current input resolution (640 × 640 pixels), insufficient pixel information was available to support reliable feature representation. Consequently, the triple-attention mechanism in the SPPF_TSFA module was unable to effectively capture the subtle lesion features. (2) Environmental factors such as water flow disturbances and low contrast in underwater conditions further diminished lesion visibility, limiting the effectiveness of the SDIoU loss function in optimizing detection for extremely small targets during scale-adaptive adjustments.

To address the challenges of detecting extremely small lesions, several potential improvements are proposed for future work: (1) employ higher input resolutions (e.g., 1280 × 1280 pixels) to enhance the amount of pixel information and improve feature extraction capabilities; (2) introduce super-resolution reconstruction techniques (e.g., SRGAN) during the preprocessing stage, to enhance lesion detail visibility; (3) optimize the SPPF_TSFA module by incorporating dedicated attention branches for small-object detection, such as finer-grained spatial attention mechanisms; and (4) integrate multi-scale feature fusion strategies to increase the model’s sensitivity to extremely small lesions. Additionally, leveraging temporal data or multi-frame analysis could exploit motion patterns of lesions across consecutive frames, thereby compensating for the limited pixel information in single-frame images. These enhancements are expected to significantly improve the YOLO-TPS model’s performance in detecting minute lesions and strengthen its applicability in early disease warning systems for aquaculture.

### 4.5. Trade-Off Analysis Between Lightweight Design and Computational Cost

The YOLO-TPS model was designed to maintain high detection accuracy while achieving a lightweight architecture, thereby facilitating deployment in resource-constrained environments. Experimental results demonstrate that YOLO-TPS contains 2.513 million parameters, slightly lower than the baseline YOLOv11n model (2.583 million parameters). The model size is 5.4 MB, marginally larger than YOLOv11n’s 5.2 MB. However, the floating-point operations (FLOPs) increased from 6.3G in YOLOv11n to 6.9G in YOLO-TPS, indicating a moderate rise in computational cost. This increase can be attributed to the integration of the following components:

(1) The SPPF_TSFA module, which employs a three-level attention mechanism to enhance multi-scale feature extraction, incurs a slight computational overhead, to improve the detection accuracy of small-scale lesions.
(2) The PC_Shuffleblock module replaces conventional 1 × 1 convolutions with turbine-shaped convolutions, effectively enlarging the receptive field and enhancing spatial modeling capability. Although its sparse connectivity design reduces parameter count, the complexity of the convolutional operations slightly increases the computational load.
(3) The SDIoU loss function, with its dynamic weighting adjustment during training, introduces additional computational complexity, aimed at optimizing the model’s adaptability to dynamic lesion features.

Despite the approximately 9.5% increase in FLOPs, YOLO-TPS exhibits substantial performance gains—mAP@0.5 increased from 93.4% to 97.2%, Precision from 92.5% to 97.9%, and Recall from 90.4% to 95.1%—demonstrating that the additional computational cost is justified. Compared to conventional models such as Faster R-CNN (207 G FLOPs, 44.297 M parameters) and SSD (37.8 G FLOPs, 30.245 M parameters), YOLO-TPS retains a significant lightweight advantage, with computational complexity and model size reduced to approximately 3.3% and 1.7% of those models, respectively. Furthermore, compared to other YOLO series models (e.g., YOLOv8n and YOLOv10n), YOLO-TPS achieves superior detection accuracy, with only a marginal increase in FLOPs. Notably, it excels in recognizing small targets and blurred lesions under complex aquaculture conditions. This balance between performance and computational cost indicates that YOLO-TPS maintains strong deployment feasibility on edge devices with limited resources (e.g., embedded systems or mobile devices), while satisfying the high-precision and real-time detection demands of aquaculture environments.

To further enhance computational efficiency, future work could explore model compression techniques such as pruning or quantization, aiming to reduce FLOPs without compromising performance [11]. Additionally, to address the challenges of detecting dynamic targets in underwater environments, spatiotemporal modeling approaches based on video sequences—such as optical flow analysis or video object detection—may be investigated to improve robustness in dynamic scenarios [19,28]. These advancements would further enhance the practical applicability of YOLO-TPS in real-world aquaculture monitoring systems.

### 4.6. Model Generalization and Application Potential

The experimental results demonstrate that YOLO-TPS achieves an average precision (mAP_0.5_) of 97.2%, representing a 3.8% improvement over YOLOv11n. Moreover, it attains substantial enhancements in both Precision (97.9%) and Recall (95.1%). Comparative evaluations against established models such as Faster R-CNN, SSD, and YOLOv5 through YOLOv10, indicate that YOLO-TPS attains the highest accuracy, while maintaining a relatively small parameter count (2.513 million) and low computational complexity (6.9 GFLOPs). These findings further substantiate the systematic performance gains conferred by the multi-module integrated architecture. This improvement aligns closely with the principle of achieving an optimal balance between model lightweightness and representational capacity, as emphasized in prior studies by Pandian et al. [4] and Ghosh et al. [12].

Additionally, Grad-CAM visualizations reveal that YOLO-TPS consistently focuses on lesion regions across diverse detection scenarios, effectively suppressing background noise interference. The model maintains robust localization consistency and prediction stability under challenging conditions, including overlapping multiple fish, complex backgrounds, and irregular lighting. Such characteristics are particularly critical for intelligent diagnostics in agricultural- and animal-disease contexts [8,10,22], and further underscore the model’s strong cross-domain generalization capability.

It is noteworthy that, as shown in Table 9, the YOLO-TPS model exhibited relatively lower performance in detecting the EUS (Epizootic Ulcerative Syndrome) class, with a Precision of 92.6%, Recall of 77.9%, and mAP@0.5 of 86.4%, which are substantially lower than the corresponding metrics for other classes such as gill disease and columnaris disease (ranging from 99.4% to 99.5%). This performance disparity may be attributed to the inherent characteristics of EUS lesions, which typically involve deep skin ulcerations that appear with high blurriness and low contrast in images. These visual properties hinder the model’s ability to accurately extract discriminative features and distinguish lesions from background noise. Moreover, the relatively limited number of EUS samples in the public dataset may further constrain the model’s learning capacity for this specific category.

To mitigate this issue, future work could explore the use of Generative Adversarial Networks (GANs) to augment the dataset by synthesizing additional EUS samples [24], or employ domain-adaptation techniques to enhance the model’s robustness in recognizing low-contrast lesion features [35]. Additionally, considering the depth and textural complexity of EUS lesions, the SPPF_TSFA module could be further optimized by incorporating depthwise separable convolution layers to improve the model’s capacity to represent blurry lesion edges, effectively.

Furthermore, the dataset used in this study encompasses six representative fish-disease categories, and was constructed as a composite dataset by integrating publicly available data from Roboflow with images collected from field investigations. This multi-source dataset enhances the model’s generalizability under heterogeneous-data conditions. Consistent with multi-class data fusion strategies reported by Li et al. [26], Yu et al. [21], and Tang et al. [27], the diversity and annotation accuracy of the dataset are critical factors underpinning the improved performance of deep learning models in complex aquatic disease detection tasks.

### 4.7. Limitations and Future Directions

Although YOLO-TPS demonstrates excellent performance in static image detection, several limitations persist in real-world aquaculture scenarios. First, the continuous movement of fish and dynamic occlusions in underwater environments can lead to discontinuities in target features across image sequences, thereby impairing the model’s real-time detection capability. To address this challenge, future research could incorporate temporal modeling techniques—such as video object detection or optical flow analysis—to capture dynamic features and enhance the model’s adaptability to moving targets [19,28]. Second, extreme lighting conditions (e.g., strong underwater reflections or low-light environments) and severe occlusions can further degrade image quality, adversely affecting lesion feature extraction. To improve robustness under such complex conditions, image augmentation techniques—such as CycleGAN-based sample generation—can be employed to expand data diversity, or multimodal data sources (e.g., infrared or ultrasound imaging) may be integrated to enhance the model’s resilience [16,24]. Third, the geographical and species-specific limitations of the current dataset may constrain the model’s generalization across different regions and fish species. Future work should consider expanding the dataset to include a wider variety of fish species and farming environments (e.g., marine aquaculture or variable water-quality conditions) to validate the broader applicability of the model.

Overfitting also presents a critical challenge for deploying deep learning models in field applications, particularly under conditions of limited sample size or imbalanced data distribution. YOLO-TPS effectively mitigated overfitting risks through data augmentation strategies (including image rotation and brightness/saturation adjustment) and regularization techniques (e.g., weight decay with a coefficient of 0.0005). The loss curves of the training and validation sets (Figure 11) exhibited consistent convergence trends, indicating no significant overfitting. However, performance fluctuations observed in categories with fewer samples, such as EUS, suggest a potential overfitting risk. To further improve generalization, techniques such as Dropout (with a probability of 0.1–0.3) or label smoothing could be introduced. Additionally, implementing k-fold cross-validation (k = 5) during training would provide a more robust evaluation of the model’s performance on unseen data. For deployment on edge devices, model pruning and quantization techniques could be employed to reduce computational complexity, thereby mitigating overfitting risks while enhancing real-time inference efficiency [11].

### 4.8. Comprehensive Performance and Cross-Domain Application Expansion

In summary, the YOLO-TPS model demonstrates superior overall performance in fish-disease detection tasks. Its multi-module collaborative strategy achieves significant improvements in model accuracy, lightweight design, robustness, and generalization capability. The core methodology is also transferable to other domains, such as plant-leaf disease detection [4,5,7] and animal disease diagnosis [1,8,9]. For instance, the introduction of a three-level attention mechanism in corn-leaf spot detection [3] or the application of scale-adaptive dynamic loss in bovine papillomatosis identification [14] exemplify such adaptations. This study not only expands the application scope of the YOLO series in intelligent aquaculture monitoring, but also offers a valuable reference framework for diverse biological disease recognition tasks, holding substantial theoretical and practical significance for real-world deployment. 

## 5. Conclusions

To address the challenges of small-object sizes, complex backgrounds, and highly variable lesion features in fish-disease detection within aquaculture environments, this study proposes YOLO-TPS, a lightweight and high-precision detection model based on an improved YOLOv11n architecture. The model incorporates the SPPF_TSFA module into the backbone to enhance multi-scale feature representation, integrates the PC_Shuffleblock module into the detection head to improve spatial modeling and texture perception, and introduces a Scale-Dynamic IoU (SDIoU) loss function to accommodate the progressive characteristics of disease manifestation. These innovations collectively mitigate the limitations of conventional object-detection models, such as low recognition accuracy, high missed-detection rates for early-stage lesions, and poor robustness in complex scenes, thereby significantly improving the practical value of the model in fish-disease detection tasks.

Ablation experiments demonstrate the individual contributions and synergistic effects of each module in improving detection performance. When all modules are integrated into the YOLOv11n baseline, the enhanced model achieves an mAP@0.5 of 97.2%, a Precision of 97.9%, and a Recall of 95.1%, representing gains of 3.8%, 5.4%, and 4.7%, respectively, over the original model. Comparative experiments with mainstream detection algorithms such as Faster-RCNN, SSD, YOLOv5, and YOLOv8n further validate that YOLO-TPS offers superior accuracy and robustness while maintaining a lightweight structure, with only 2.513 million parameters and a model size of 5.4 MB. These features make the model well-suited for deployment in resource-constrained environments. Feature visualization analysis reveals that YOLO-TPS can more precisely focus on key lesion areas, demonstrating stronger feature-discrimination and spatial-localization capabilities. Notably, the model maintains stable performance across various background conditions, underscoring its potential for early-stage disease detection and accurate diagnosis in real-world aquaculture settings.

In terms of data support, this study constructed a high-quality, balanced, and representative fish-disease image dataset that includes both healthy and Saprolegniasis-infected samples collected from aquaculture bases, as well as five common fish diseases from the publicly available Roboflow dataset. A unified annotation protocol and data augmentation strategy were employed to increase the diversity and complexity of training samples, thereby enhancing the generalization capacity of the deep learning model. Despite the dataset’s strengths in class diversity and environmental variability, certain disease categories remain under-represented. Future work will focus on expanding the dataset to include a wider range of disease types and geographic regions, thereby improving the model’s cross-domain robustness and practical deployment potential.

In summary, the systematic improvements in architecture, loss function design, and feature enhancement embodied in the YOLO-TPS model provide an efficient, accurate, and deployable solution for fish-disease detection. This research not only advances the technological foundation for automated aquatic animal health monitoring but also contributes to the broader goal of sustainable and welfare-oriented aquaculture by supporting early disease warning, minimizing antibiotic use, and optimizing production system management. The approach and findings presented here have strong potential for adaptation to other species and disease contexts, providing valuable insights for future innovations in animal health management and precision livestock systems.

## Figures and Tables

**Table 1:** Fish-disease data collection details.

Shooting Equipment	Shooting Angle	Pixel Size	Shooting Category
IQOO NEO7	Top	4096 × 2304	Saprolegniasis
IQOO NEO7	Bottom	4096 × 2304	Saprolegniasis
IQOO NEO7	Side	4096 × 2304	Saprolegniasis
VIVO X100 Pro	Side	4096 × 3072	Healthy

**Table 2:** Sample quality-control strategies.

Inspection Dimension	Description of Control Criteria	Inclusion in Dataset
Image Blurriness	Laplacian operator variance >100, main fish body structure identifiable by naked eye, edges not completely blurred	Yes
Lighting Conditions	Brightness mean within [50, 200], partial reflection allowed, avoiding complete overexposure/underexposure	Yes
Annotation Completeness	At least one target region annotated per image, accurate category labeling, Kappa coefficient >0.85	Yes
Multi-fish Overlapping Scenarios	Acceptable if ≥50% of fish body contour is clearly visible, completely overlapped or fishless images excluded	No

**Table 3:** Image Data Augmentation.

Category	Number of Annotation Boxes (Before Augmentation)	Number of Annotation Boxes (After Augmentation)
Healthy Fish	245	490
Saprolegnia infection Fish	304	608
Total	549	1098

**Table 4:** Dataset composition statistics.

Data Source	Number of Categories	Number of Images	Original Resolution	Annotation Tool	Annotation Format	Augmented
Self-collected Data	two categories (healthy, Saprolegniasis)	1068 (after augmentation)	4096 × 2304	LabelMe	YOLO format	Yes
Public Dataset	five categories (including healthy)	3528 (after original augmentation)	640 × 640	Roboflow platform	YOLO format	Yes
Total	six categories	4596	Uniformly adjusted to 640 × 640	—	YOLO format	Yes

**Table 5:** Model training parameters.

Parameter	Set Value
Model Training Epochs	100
Batch Size	16
Initial Learning Rate	0.01
Optimizer	SGD
Momentum	0.937
Input Image Resolution	640 × 640
Weight Decay Coefficient	0.0005
Random Seed	42, 123, 645

**Table 6:** PC_Shuffleblock module insertion-position comparison.

Experimental Group	Insertion Position (Layer Index)	mAP_0.5_ (%)	Precision (%)	Recall (%)
A	P0/P1	95.2	96.2	92.0
B	P5/P7	94.7	94.0	92.2
C	P3/P5	94.1	94.3	89.7
D	P7/P17/P20	95.5	95.1	93.4
E	P17/P20	97.2	97.9	95.1

**Table 7:** Ablation-experiment Performance Comparison.

YOLOv11n	SPPF_TSFA	PC_Shuffleblock	SDIoU	mAP_0.5_ (%)	Precision (%)	Recall (%)
√				93.4	92.5	90.4
√	√			94.3	93.8	90.7
√		√		95.1	95.1	92.8
√			√	94.7	94.2	91.7
√	√	√		95.6	95.3	93.2
√	√		√	95.4	94.4	93.4
√		√	√	95.2	95.6	91.7
√	√	√	√	97.2	97.9	95.1

**Table 8:** Comparison of different models’ experimental results.

Model	Parameters/M	FLOPs/G	Model Size/MB	mAP_0.5_ (%)	Precision (%)	Recall (%)
Faster-RCNN	44.297	207	315	61.2 ± 0.8	70.5 ± 0.7	65.9 ± 0.9
SSD	30.245	37.8	104	56.2 ± 0.9	53.1 ± 1.0	44.2 ± 1.0
YOLOv5n	1.767	4.2	3.7	90.2 ± 0.6	87.4 ± 0.5	86.9 ± 0.7
YOLOv8n	3.006	8.1	5.9	91.4 ± 0.5	91.6 ± 0.4	90.3 ± 0.6
YOLOv9n	2.803	11.7	23.4	92.3 ± 0.6	88.8 ± 0.4	89.0 ± 0.5
YOLO10n	2.696	8.2	5.7	92.2 ± 0.5	91.5 ± 0.5	89.1 ± 0.6
YOLO11n	2.583	6.3	5.2	93.4 ± 0.4	92.5 ± 0.4	90.4 ± 0.5
**YOLO-TPS** **(** **Ours** **)**	**2.513**	**6.9**	**5.4**	**97.2 ± 0.3**	**97.9 ± 0.2**	**95.1 ± 0.3**

**Table 9:** Results of the YOLO-TPS model for different fish categories.

Class	Precision (%)	Recall (%)	mAP_0.5_ (%)
All	97.9	95.1	97.2
EUS	92.6	77.9	86.4
Gill Disease	99.3	97.9	99.4
Columnaris Disease	99.3	98.1	99.5
Streptococcus Disease	98.6	98.7	99.1
Saprolegniasis	98.9	99.5	99.5
Healthy	98.4	98.3	99.1

## Data Availability

Data are available on request, due to privacy.
